# Honokiol Ameliorates DSS-Induced Mouse Colitis by Inhibiting Inflammation and Oxidative Stress and Improving the Intestinal Barrier

**DOI:** 10.1155/2022/1755608

**Published:** 2022-12-19

**Authors:** Lu Wang, Junping Wang

**Affiliations:** ^1^Department of Gastroenterology, Second Hospital of Shanxi Medical University, Taiyuan, China; ^2^The Fifth Clinical Medical College of Shanxi Medical University, Taiyuan, Shanxi, China; ^3^Department of Gastroenterology, Fifth Hospital of Shanxi Medical University, Taiyuan, Shanxi, China

## Abstract

Ulcerative colitis (UC) is a multifactor intestinal disease with increased morbidity. Recently, pleiotropic drugs with exact biosafety have been urgently needed. Honokiol (HKL) is the major bioactive component of traditional Chinese medicine “Houpu,” with almost no toxic effects and approved anti-inflammation, antioxidant, antispasmodic, etc. effects. This study examined the therapeutic effect of HKL in dextran sulfate sodium- (DSS-) induced experimental colitis. In vivo, C57BL/6 mice received 3% DSS for seven days to generate UC, and HKL was pretreated for five days and given during the whole DSS-induced period. In vitro, RAW264.7 macrophages were stimulated with lipopolysaccharide (LPS) to induce inflammation, and mouse colon epithelial cells (MCEC) were treated with HKL or pretreated with HKL and then stimulated with LPS-induced macrophage supernate to investigate the barrier enhancement roles. HKL significantly ameliorated disease activity index (DAI), colon length, and histopathological scores in DSS-induced colitis. The inflammatory mediators of interleukin 1*β* (IL-1*β*), interleukin 6 (IL-6), tumor necrosis factor *α* (TNF-*α*), inducible nitric oxide synthase (iNOS), and cyclooxygenase-2 (COX2) were decreased, and the tight conjunction proteins were increased in the HKL-treated group both in vivo and in vitro. Above all, HKL can relieve experimental UC through anti-inflammation, antioxidant, and epithelial barrier enhancement roles. These effects were associated with peroxisome proliferator-activated receptor *γ* (PPAR*γ*)/nuclear factor-*κ*B (NF-*κ*B) p65, sirtuin3 (SIRT3)/adenosine 5′-monophosphate- (AMP-) activated protein kinase (AMPK), and nuclear factor erythroid 2-related factor 2 (NRF2)/heme oxygenase 1 (HO1) signaling pathways. In conclusion, after further clinical studies, HKL may be a promising drug for UC.

## 1. Introduction

UC is a chronic relapsing inflammatory disease limited to the mucosal layer of the colon [1]. The exact pathogenesis of UC is still unknown; the most widely accepted view is that it is multifactorial, including dysregulated immune responses, altered gut microbiota, genetic susceptibility, and environmental factors [1–3]. The global burden of UC continues to increase, for instance, in the US market, from $5.0 billion in 2020 to predicated $11-12 billion by 2026 [4]. The new drugs for UC treatment have been impressive in recent years. However, none is effective in most patients, let alone the biologic-experienced patients [5]. In a nationwide study in Israel, colectomy and steroid dependency rates have remained unchanged in patients with UC during the biological era [6]. The large proportion of primary nonresponders to each molecularly targeted agent may be due to different dominant cytokine levels depending on disease stage and patient characteristics [7]. The pathology of UC is very complex. Up to now, there is no treatment and unlikely to have a therapy suitable for all. So, there is an urgent need for low cost, few side effects, and pleiotropic drugs to enrich current treatment options.

**Scheme 1:**
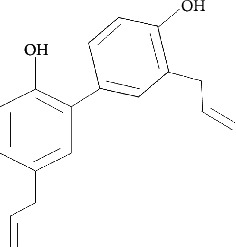
Structure of honokiol (HKL).

HKL is a simple biphenyl neolignane found in Magnolia species [8]. Its structure is shown in Scheme 1. It is brown to white fine powder, aromatic, spicy taste, and slightly bitter. It is soluble in general organic solvents; easily in benzene, ether, chloroform, ethanol, etc.; and insoluble in water [9]. It is a potent bioactive compound of traditional Chinese medicine “Houpu.” According to the Chinese medicine meridian theory, “Houpu” returns to the large intestine meridian and is widely used to treat gastrointestinal diseases. Modern investigations have confirmed that HKL has anti-inflammation [10], antimicrobial [11, 12], antioxidant [13–15], antispasmodic [16], anticancer [8, 17–19], antithrombotic [20], antidepressant, anxiolytic [21], and other actions [22, 23]. As known to all, synthetic drugs have many side effects, including damage to the endocrine system, immune system, and gastrointestinal tract. Regarding the numerous pharmacologic benefits without noticeable side effects of HKL, we hypothesize that HKL might have therapeutic effects on UC.

In the present study, we investigated the anti-inflammation, antioxidant, and intestinal barrier enhancement roles of HKL on UC treatment in vitro and in vivo. The mechanisms responsible for its actions have also been explored.

## 2. Materials and Methods

### 2.1. Animal Model and Treatments

Six- to eight-week-old female C57BL/6 mice weighed 15-20 g and were purchased from the Laboratory animal center of Shanxi Provincial People's Hospital (Shanxi, China, animal license number: SYXK (Jin) 2019-0003) and kept in the specified pathogen-free (SPF) condition at 22-23°C with a standard 12 h light/dark cycle. All mice have free access to food and water. Forty mice were randomly divided into four groups (*n* = 10 per group). The control group was given a normal diet; the HKL group received HKL (40 mg/kg wt. purity≥98%, Yuanye, China) dissolved in olive oil (Yuanye, China) via oral gavage (10ul/g) from D1 to D12; the DSS group received DSS (3% w/v, g/ml, 36-50 kDa, Yeasen, China) via drinking water D6-D12; the DSS+HKL group pretreated with HKL for five days and then received 3% DSS for seven days with HKL gavage once a day. The control and DSS groups received olive oil (10ul/g) once daily by gavage during the experimental period. The Animal Ethics Committee approved all animal procedures of the Second Hospital of Shanxi Medical University (Approval No. DW2022051).

### 2.2. Macroscopic Grading and Histological Analysis of Colitis

The macroscopic grading of DSS-induced colitis is based on a standard scoring system called the disease activity index (DAI) [24]. The details are shown in Table 1. The body weight, stool consistency, and rectal bleeding of each mouse were recorded in fact daily. All mice were anesthetized and sacrificed by cervical dislocation 24 h after the last drug treatment. Their colons were excised, and the length was measured promptly. The distal colonic tissues were fixed in 4% paraformaldehyde solution (Servicebio, China), embedded in paraffin (Solarbio, China), sectioned, and then stained with hematoxylin and eosin (HE) (Solarbio, China) for optical microscopic (CKX53, Olympus, Japan) observation. Histopathological scores were determined according to the previous criteria [25] shown in Table 2.

### 2.3. Immunohistochemistry

After dewaxing the paraffin-embedded colon sections, citric acid (Solarbio, China) was used for antigen reparation. Then, the slices were rinsed with 3% H_2_O_2_ (Solarbio, China) for 25 min at room temperature to block endogenous peroxidase activity and incubated with 3% bovine serum albumin (Solarbio, China) for 30 min to block nonspecific binding. Followed by setting with the first antibodies (Proteintech, USA) in a wet box at 4°C overnight, the sections were incubated with the corresponding secondary antibody: anti-rabbit IgG antibody conjugated to horseradish peroxidase (1 : 200) (Proteintech, USA). Then, the freshly prepared diaminobenzidine (DAB) (Solarbio, China) chromogenic solution was dripped on the slices for coloration. The coloration time was controlled under a microscope (CKX53, Olympus, Japan), rinsing with tap water to stop. Counterstaining was performed using HE (Solarbio, China), sealing the sections with synthetic resin (Servicebio, China). Finally, we conducted the microscope examination and the positive results were shown in brown.

### 2.4. Cell Culture

RAW264.7 macrophages, purchased from Bena Culture Collection, and MCEC, purchased from Bluefbio, were both cultured in Dulbecco's Modified Eagle Medium (DMEM) (Gibco, Australia) containing 10% fetal bovine serum (FBS) (Gibco, Australia), maintaining at 37°C in a humidified chamber (HF100, Heal Force, China) of 5% CO_2_. The cells were seeded into a 10 cm plate and subcultured every 2-3 days when the cells were 90% confluence. For further experiments, cells were seeded in a 6-well plate and treated with HKL or LPS (Solarbio, China) when the RAW264.7 cells were 80% confluence and MCEC 100% confluence, respectively.

### 2.5. Cell Viability Assay

RAW264.7 cells or MCEC were seeded into 96-well plates (NEST, China) (10^3^-10^4^ cells/well) and treated with various concentrations of HKL for 24 h. Then, 10ul of Cell Counting Kit-8 (CCK-8) (Boster, China) was added to each well for reaction; 15-30 minutes later, a microplate reader (Epoch, BioTek, USA) was used to measure the OD values of each well at 450 nm. The cellular viability was calculated following the instructions of the manufacturer.

### 2.6. Transepithelial Electric Resistance (TEER)

**Figure 1:**
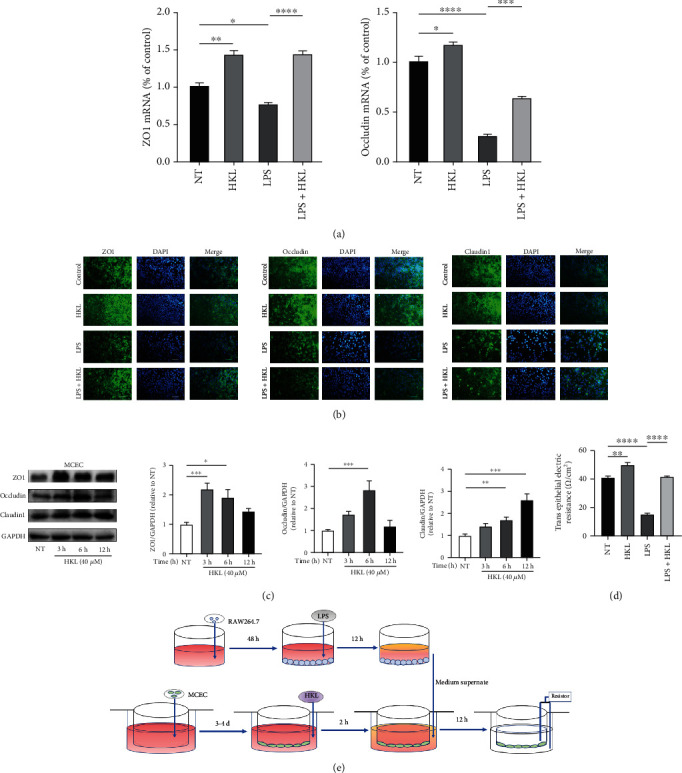
HKL maintained a normal epithelium barrier in MCEC. (a) MCEC were pretreated with 40 *μ*M HKL for one hour and then stimulated with the supernatant of 12h-LPS-induced RAW264.7 cells for 12 h. QRT-PCR analyses measured the mRNA expression of ZO1 and occludin; mRNA expression was normalized to *β*-actin expression. (b) Cell immunofluorescence of tight junction proteins in confluent MCEC pretreated with 40 *μ*M HKL for one hour and then stimulated with the supernatant of 12h-LPS-induced RAW264.7 cells for 12 h. Fluorescent microscopy obtained representative images from three independent experiments after immunofluorescence staining of ZO-1, occludin, and Claudin1. DAPI staining was performed to identify nuclei. (c) MCEC were grown to confluence on 6-well plates and treated with HKL (40 *μ*M) for the indicated periods. Western blots and related quantification of ZO-1, occludin, and Claudin1 are expressed as a percent of control. (d) TEER of various treatments of the MCEC monolayer. (e) Schematic diagram of the testing process of TEER. Data are expressed as mean ± SEM (*n* = 3). ^∗^*P* < 0.05, ^∗∗^*P* < 0.01, ^∗∗∗^*P* < 0.001, and ^∗∗∗∗^*P* < 0.0001.

The MCEC were well suspended and seeded at a density of 10^5^ cells/well, 0.5 ml suspension was added to the upper chamber of a Transwell plate (12 mm diameter inserts and 0.4 um pore size) (Corning Costar, USA), and 1.5 ml of culture medium was added to the lower chamber, cultured for 3-4 days to form a cell monolayer. The culture medium was replaced with an equal volume of D-Hank's solution (Boster, China) and incubated for 20 min at 37°C. Then, the resistance value was acquired by using an epithelial voltmeter (EVOM^2^, WPI, USA) and inserting two silver chloride electrodes on both sides of the Transwell membrane. The resistance values were measured every other day. When the values were stable, the LPS group has added the supernatant of 12h-LPS-induced RAW264.7 macrophages; the HKL group was added HKL (40 uM) for 14 h; the HKL+LPS group was added HKL (40 uM) for 2 h, then added the supernatant of 12h-LPS-induced RAW264.7 cells for another 12 h, respectively, to the lower chamber. The details are shown in Figure 1(e). After that, the resistance value of each group was measured as mentioned above. The TEER calculation formula is as follows: TEER = (*R* − RB) × surface area (*R* is the resistance value of the testing well, RB is the resistance value of the blank well, and surface area = 1.12 cm^2^).

### 2.7. Cell Immunofluorescence

When corresponding conditions had treated cells, they were washed with 37°C 1x PBS (Meilunbio, China) twice, then fixed with 4% paraformaldehyde (Servicebio, China) for 10 min, and washed with 1x PBS thrice. Then, incubate with 0.1% Triton X-100 (Mini Bio, China) for 15 min and washed with 1x PBS thrice. They were blocked with 5% bovine serum albumin (Solarbio, China) for one hour at room temperature. Then, cells were incubated with the primary antibodies against NF-*κ*B p65 (Proteintech, USA), NRF2 (Proteintech, USA), ZO1 (Proteintech, USA), occludin (Proteintech, USA), and Claudin1 (Proteintech, USA) diluted to 1 : 500 at 4°C overnight and washed with cold PBS sufficiently thrice. Then the cells were incubated with secondary antibodies (ABclonal, USA) (1 : 500) at room temperature for one hour and washed with PBS thrice. Subsequently, cells were stained with 4′,6-diamidino-2-phenylindole (DAPI) (Solarbio, China) for 5 min. The images were acquired using a Nikon Ni-U upright fluorescence microscope.

### 2.8. Western Blot Analysis

Proteins were extracted from both colon tissue homogenates and cells using RIPA lysis buffer (Solarbio, China) containing 1% phenylmethyl sulfonyl fluoride (Solarbio, China) and 1% protein phosphatase inhibitors (Solarbio, China), centrifuging at 12000g at 4°C for 15 min (Centrifuge 5430R, Eppendorf, Germany), and collecting the suspension liquid. The protein concentration was determined using a bicinchoninic acid kit (Thermo Fisher, USA). Equal amounts of protein extract samples (40-50 *μ*g) were separated by 10% or 12% sodium dodecyl sulfate- (SDS-) polyacrylamide gel electrophoresis and transferred onto a polyvinylidene difluoride (PVDF) membrane (Immobilon-P^SQ^, Millipore, Ireland). After blocking the PVDF membranes with 5% skim milk (Solarbio, China) for one hour at room temperature, washed with Tris-buffered saline Tween (TBST) solution twice, the membranes were incubated with the primary antibodies (NF-*κ*B p65 (Proteintech, USA), NRF2 (Proteintech, USA), HO1 (Proteintech, USA), phosphorylation-AMPK*α* (Thr172) (Cell Signaling, USA), AMPK (Proteintech, USA), SIRT3 (Proteintech, USA), COX2 (Proteintech, USA), iNOS (Proteintech, USA), ZO1 (Proteintech, USA), occludin (Proteintech, USA), Claudin1 (Proteintech, USA), and GAPDH (Proteintech, USA) (dilution 1 : 1000)) overnight at 4°C and washed with TBST thrice, followed by incubation with an HRP-conjugated anti-mouse or anti-rabbit secondary antibody (ABclonal, USA) (dilution 1 : 2000) at room temperature for one hour and rinsed thrice with TBST buffer. Finally, protein bands were reacted to an enhanced chemiluminescence solution (Boster, China) and detected using the Bio-Rad Image Lab system (ChemiDoc XRS+, Bio-Rad, USA). The intensity of the bands was quantified using the ImageJ software, and the results of GAPDH normalized the relative protein expression levels.

### 2.9. Real-Time Quantitative Polymerase Chain Reaction (qRT-PCR)

Total RNA was extracted from the colon tissue homogenates, and RAW264.7 cells or MCEC using RNAiso Plus (Takara, Japan) and their concentrations were determined. The primer sequences of interleukin IL-1*β*, IL-6, TNF-*α*, ZO1, occludin, and Claudin1 are shown in Table 3. 1 *μ*g RNA was reverse-transcribed into complementary DNA using a commercial RT-PCR kit (Takara, Japan). RT-PCR assays were performed using the 2x M5 HiPer SYBR Green qPCR SuperMix (Mei5 Biotechnology, China) with the CFX96 Real-Time PCR Detection System (CFX Connect, Bio-Rad, USA). Each sample was analyzed in triplicate. The expression levels of target mRNA were normalized with *β*-actin and calculated with the 2^-*ΔΔ*ct^ method.

### 2.10. Statistical Analysis

All data are shown as mean ± SEM. The statistical analyses were performed in the GraphPad Prism software version 8.0. The significance of the differences between groups was analyzed by one-way ANOVA followed by Tukey's multiple range tests. *P* < 0.05 was regarded as statistically significant. For every result in this study, equal or greater than three biological repetitions were conducted.

## 3. Results

### 3.1. HKL Ameliorated DAI and Colon Length in DSS-Induced Colitis in Mice

**Figure 2:**
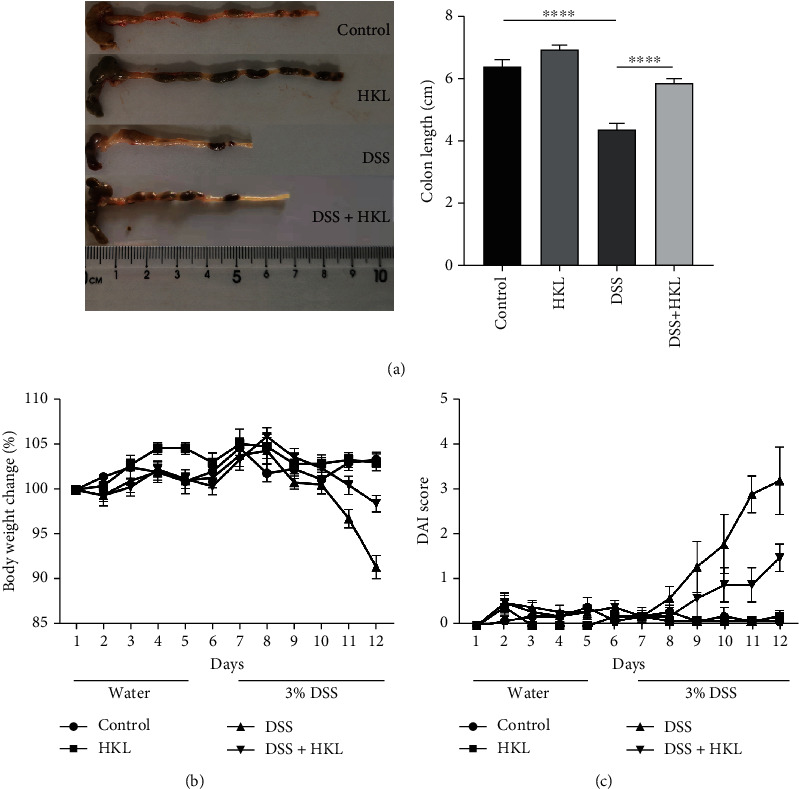
HKL ameliorated DAI and colon length in DSS-induced colitis in mice. (a) Colon length; (b) body weight change; (c) DAI. Data are expressed as mean ± SEM (*n* = 10). ^∗∗∗∗^*P* < 0.0001 and ^∗∗∗^*P* < 0.001 versus control; ^####^*P* < 0.0001, ^###^*P* < 0.001, and ^#^*P* < 0.05 versus DSS alone.

DSS-induced experimental colitis is a classical ulcerative colitis animal model for its similar symptoms and histopathological characteristics. In this study, the HKL treatment group had lower disease activity index, which consists of weight loss, stool character, and fecal occult blood, compared with the DSS group (Figure 2(c)). Among these indexes, the body weight change is a quantifiable visual indicator of the state of the mice. As shown, the body weight of the model group mice decreased remarkably from the fifth day when they received special water containing 3% DSS. In contrast, the HKL pretreated group showed a mitigated decrease in body weight (Figure 2(b)). The shortening of colon length revealed the severity of DSS-induced colitis. The HKL+DSS group showed an attenuated degree of this trend compared with the DSS group (Figure 2(a)).

### 3.2. HKL Treatment Decreases Inflammatory Mediators in DSS-Induced Mice

**Figure 3:**
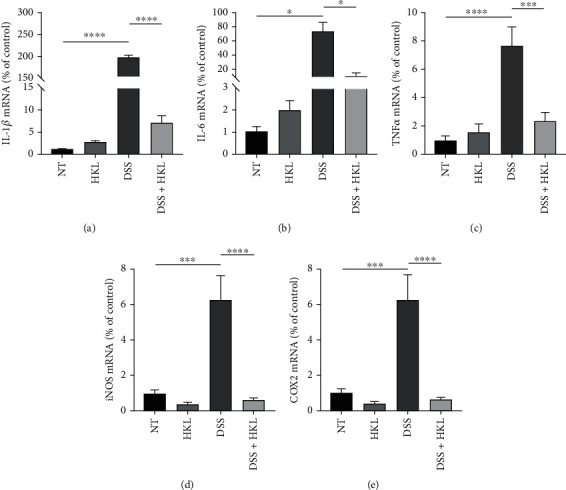
HKL treatment decreases inflammatory mediators in DSS-induced mice. The levels of (a) IL-1*β*, (b) IL-6, (c) TNF-*α*, (d) iNOS, and (e) COX2 mRNA were measured by qRT-PCR analyses. Data are expressed as mean ± SEM (*n* = 3). ^∗∗^*P* < 0.01, ^∗∗∗^*P* < 0.001, and ^∗∗∗∗^*P* < 0.0001.

The qRT-PCR results showed that the levels of IL-1*β* (Figure 3(a)), IL-6 (Figure 3(b)), TNF-*α* (Figure 3(c)), iNOS (Figure 3(d)), and COX2 (Figure 3(e)) significantly increased in colon tissue homogenates of the DSS group compared with the control group. In contrast, the levels of the above inflammatory mediators were marked decreased in the HKL treatment group compared with the DSS group. These data suggested that HKL played an anti-inflammatory role in vivo.

### 3.3. HKL Treatment Improved the Histopathological Features of the DSS-Induced Mice

**Figure 4:**
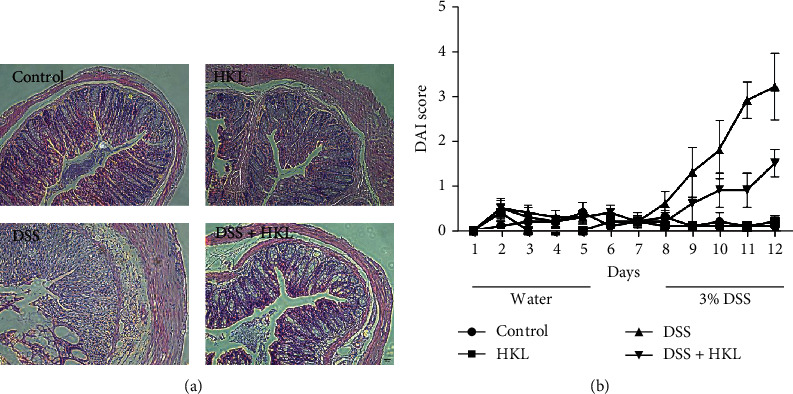
HKL treatment improved the histopathological features of the DSS-induced mice. (a) Representative images of HE staining (×200). (b) Histopathological score. Data are expressed as mean ± SEM (*n* = 10). ^∗∗∗∗^*P* < 0.0001.

The histopathological features of the HE-stained sections showed marked infiltration of inflammatory cells, loss of crypts, formation of crypt abscesses, destruction of the mucosal layer, and thickening of the muscular layer in the DSS group. In contrast, the DSS+HKL group showed alleviated pathological injuries of the above features (Figure 4(a)). This is also measured by the histological score to show more intuitively (Figure 4(b)). HKL could mitigate the histopathological injuries of experimental colitis from this study, which is also the golden standard of clinical cure.

### 3.4. HKL Improved the Barrier Function of the DSS-Induced Mice

**Figure 5:**
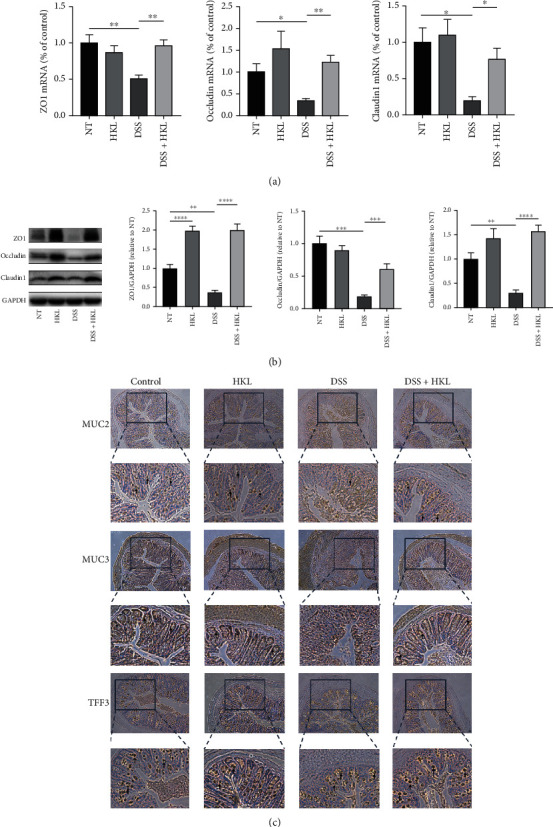
HKL improved the barrier function of the DSS-induced mice. (a) The levels of ZO1, occludin, and Claudin1 mRNA were measured by qRT-PCR analyses. mRNA expression was normalized to *β*-actin expression. (b) Western blots and related quantification of ZO1, occludin, and Claudin1 are expressed as a percent of control. (c) Immunohistochemistry analysis of the expression of MUC2, MUC3, and TFF3 (×200); the black arrows mark the protein. Data are expressed as mean ± SEM (*n* = 3). ^∗^*P* < 0.05, ^∗∗^*P* < 0.01, ^∗∗∗^*P* < 0.001, and ^∗∗∗∗^*P* < 0.0001.

The gene-expressed levels (Figure 5(a)) and the protein-expressed levels (Figure 5(b)) of ZO1, occludin, and Claudin1 increased dramatically in the DSS+HKL group compared with the DSS group. Additionally, the colon tissue immunohistochemistry showed the expression of MUC2, MUC3, and trefoil factor-3 (TFF3) decreased in DSS-induced mice; however, HKL treatment ameliorated the levels of the above proteins (Figure 5(c)). These results indicated that HKL improved barrier function in DSS-induced colitis partially by restoring the tight junction proteins, mucins, and TFF3 levels.

### 3.5. HKL Suppressed Proinflammatory Cytokine Expression in LPS-Stimulated Macrophages

**Figure 6:**
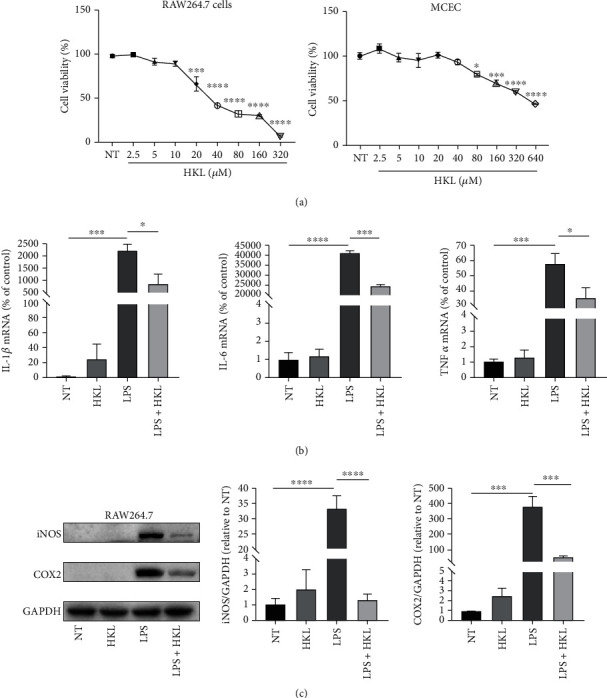
HKL suppressed proinflammatory cytokine expression in LPS-stimulated macrophages. (a) CCK-8 analyzed the effects of HKL at different concentrations on the viability of RAW264.7 cells and MCEC. (b) RAW 264.7 cells were pretreated with 10 *μ*M HKL for one hour and then stimulated with LPS (1 *μ*g/ml) for 6 hours. QRT-PCR analyses of the mRNA expression of IL-1*β*, IL-6, and TNF-*α* were measured. mRNA expression was normalized to *β*-actin expression. (c) RAW 264.7 cells were pretreated with 10 *μ*M HKL for one hour and then stimulated with LPS (1 *μ*g/ml) for 12 h. Western blots and related quantification of iNOS and COX2 expressed as a percent of control. Data are expressed as mean ± SEM (*n* = 3). ^∗^*P* < 0.05, ^∗∗∗^*P* < 0.001, and ^∗∗∗∗^*P* < 0.0001.

Cell viability assay showed that for RAW264.7 cells, HKL did not affect cell viability up to 10 *μ*M; for MCEC, the concentration was up to 40 *μ*M for no significant difference (Figure 6(a)); combined with previous studies [22], 10 *μ*M and 40 *μ*M concentrations were chosen for RAW264.7 cells and MCEC, respectively, for subsequent experiments. The qRT-PCR analyses indicated that the proinflammatory cytokines, including IL-1, IL-6, and TNF-*α*, increased markedly in 6h-LPS-induced RAW264.7 cells (Figure 6(b)), and the western blot analyses showed that the expression of iNOS and COX2 also significantly increases in 12h-LPS-induced RAW264.7 cells (Figure 6(c)). In contrast, the LPS+HKL group showed reduced gene and protein expression levels of the above inflammatory mediators. Collectively, these data demonstrated that HKL had an inhibiting effect on inflammatory response in vitro.

### 3.6. HKL Maintained a Normal Epithelium Barrier in MCEC

To investigate the protective role of HKL on the intestinal epithelium barrier in vitro, the MCEC were stimulated with the supernatant of 12h-LPS-induced RAW264.7 cells. The qRT-PCR data indicated that compared with the LPS-stimulated supernatant group, the HKL pretreated group had an average level of the ZO1 and occludin mRNA expression (Figure 1(a)). Immunofluorescence was also used to show more intuitively the ZO1, occludin, and Claudin1 protein expression levels of the MCEC with the same cell stimulation condition (Figure 1(b)). In addition, the western blot analysis showed that the ZO1, occludin, and Claudin1 protein expressions were significantly increased with the HKL treatment compared with the control group. However, the optimum effects occurred at the distinct time point for different proteins (Figure 1(c)).

A Transwell plate was also used to observe the TEER change. The data showed that the supernatant of 12h-LPS-induced RAW264.7 cells significantly decreased the resistance value of the MCEC monolayer, whereas pretreating with HKL observably inhibited this reducing effect (Figure 1(d)).

Above all, these data demonstrated that HKL exerts a strengthening and repairing role in the intestinal epithelium barrier of MCEC.

### 3.7. HKL Regulated DSS-Induced Activation of PPAR*γ*/NF-*κ*B Signaling in Mice

**Figure 7:**
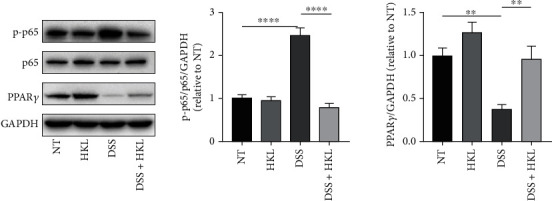
HKL regulated DSS-induced activation of PPAR*γ*/NF-*κ*B signaling in mice. Western blot analysis of protein expression levels of the phosphorylation of NF-*κ*B p65 and PPAR*γ* in colon tissue. Data are expressed as mean ± SEM (*n* = 3). ^∗^*P* < 0.05 and ^∗∗^*P* < 0.01.

It is well known that NF-*κ*B is a crucial nuclear transcription factor that regulates inflammatory response. So, the effect of HKL on phosphorylation levels of p65 in colonic tissue homogenates was analyzed using a western blot. Compared to the control group, the DSS treatment group caused elevated phosphorylation levels of p65. The DSS+HKL group showed markedly reduced phosphorylation of p65 protein. Regarding the PPAR*γ* signaling pathway that is essential for NF-*κ*B activation [26], HKL has been proven to be a PPAR*γ* agonist [27]. The expression of PPAR*γ* was further investigated. As shown in this study, PPAR*γ* significantly reduced in the colonic tissue of the DSS group, while the HKL treatment could recover this trend. Besides, HKL alone could stimulate the expression of PPAR*γ* (Figure 7).

### 3.8. The Effects of HKL on PPAR*γ*/NF-*κ*B p65, SIRT3/AMPK, and NRF2/HO1 Signaling Pathways In Vitro

**Figure 8:**
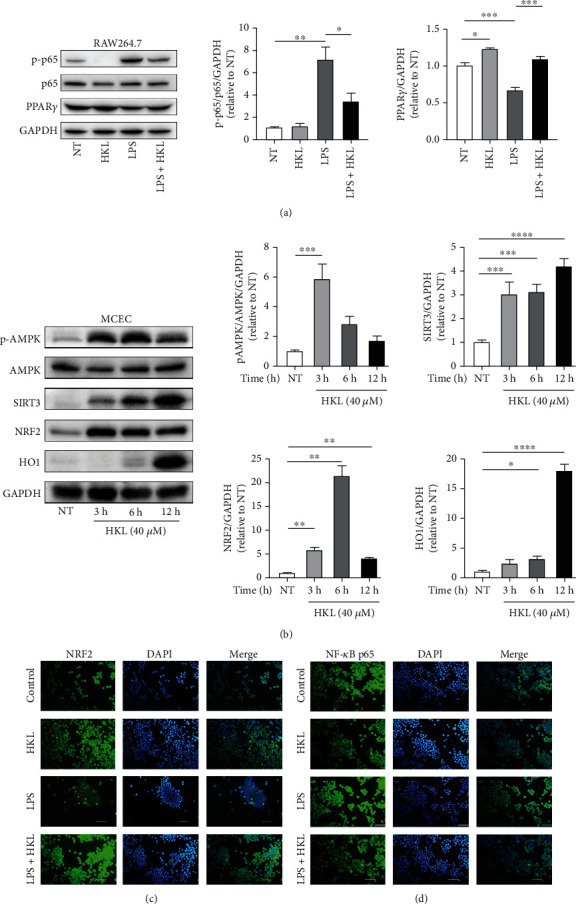
The effects of HKL on PPAR*γ*/NF-*κ*B p65 and AMPK/SIRT3/NRF2/HO1 signaling pathways in vitro. (a) RAW264.7 cells were treated with HKL (10 *μ*M) for one hour, followed by stimulation with LPS (1 *μ*g/ml) for 12 h. Western blots were used to determine the phosphorylation of the NF-*κ*B p65 signaling pathway and the expression level of PPAR*γ*. (b) MCEC were stimulated with HKL (40 *μ*M) for 3, 6, and 12 h. Total protein was extracted from the HKL-treated cells and used to determine the phosphorylation of the AMPK signaling pathway and the expression level of SIRT3, NRF2, and HO-1. (c) HKL (10 *μ*M) was used to treat RAW264.7 cells for one hour, followed by stimulating with LPS (1 *μ*g/ml) for six hours. Cell immunofluorescence analyzes the nuclear translocation of the NRF2 signaling pathway; magnification is ×200. (d) RAW264.7 cells were treated with the same conditions as the NRF2 signaling pathway. Cell immunofluorescence was performed to evaluate nuclear translocation of the NF-*κ*B p65 signaling pathway; magnification is ×200. Data are expressed as mean ± SEM (*n* = 3). ^∗^*P* < 0.05, ^∗∗^*P* < 0.01, ^∗∗∗^*P* < 0.001, and ^∗∗∗∗^*P* < 0.0001.

Similar to the findings in vivo, HKL treatment markedly reduced the elevated phosphorylation level of p65 and normalized the reduced level of PPAR*γ* (Figure 8(a)). To further explore the underlying mechanism of the epithelial barrier reinforced the role of HKL, the SIRT3/AMPK and NRF2/HO1 signaling pathways were investigated. As shown in the present study, the phosphorylation level of AMPK at Thr172, SIRT3, NRF2, and HO1 expressions was significantly increased at the different time points in MCEC, which were treated by HKL (Figure 8(b)).

Immunofluorescent staining was also used to investigate the nuclear translocation of p65 and NRF2. When the RAW264.7 macrophages were grown to 80% confluence, pretreated with 10 *μ*M HKL for one hour, and then stimulated with LPS (1 *μ*g/ml) for six hours, images showed that LPS blocked the nuclear translocation of NRF2, while HKL compensated for this effect (Figure 8(c)). In contrast, LPS significantly stimulated the nuclear translocation of NF-*κ*B p65, while HKL inhibited this process (Figure 8(d)).

## 4. Discussion

Nowadays, UC has been considered more than a superficial disease confined to the mucosa, which includes the risk of proximal extension, reduced rectal compliance, increased risk of neoplasia, reduced likelihood of responding to medical therapies, fibrosis in the submucosa, altered motility, overlapping functional symptom effects on the myenteric plexus, and even increased risks of cardiovascular morbidity [28]. With all this in-depth knowledge of UC, new individualized treatment is urgently needed. At present, histo-endoscopic remission might be a complete definition of mucosal healing, which is a desirable therapeutic goal in UC [29]. HKL showed a remarkable effect on clinical presentations and histopathological recovery of DSS-induced UC in the present study. Furthermore, chemically induced models mimic some key immunological and histopathological features of IBD in humans. The strengths of the acute models are the similar alterations of barrier function, innate immune effects, and flares [24]. So, the current results provided potential therapeutic strategies for UC in a preclinical setting. However, further investigations about HKL on the chronic models of UC should be carried on. Previously, HKL has been proven to affect stem cell viability to inhibit colon tumorigenesis by suppressing oncogenic YAP1 function [30]. In that study, an AOM/DSS-induced colitis-associated cancer model was used.

Regardless of the unclear etiology of UC, the ultimate symptom is barrier disruption, so restoration of intestinal barrier function is the primary therapeutic aim, which can be achieved by promoting inflammation resolution and accelerating mucosa reparation [2, 31]. Consequently, both the anti-inflammation and the intestinal mucosa reparation roles of HKL had been observed in this study. On the anti-inflammation aspect, inflammatory mediators, such as iNOS and COX2, and inflammatory genes, such as IL-1*β*, IL-6, and TNF-*α*, were increased in the LPS-treated RAW264.7 macrophages, and the LPS+HKL group showed decreased levels. Similar effects were also observed in the DSS and DSS+HKL in vivo groups. The anti-inflammatory effects may work through PPAR*γ*/NF-*κ*B signaling pathway. The peroxisome proliferator-activated receptor *γ* (PPAR*γ*) is a nuclear receptor highly expressed in the colon. It has been proposed as a critical inhibitor of colitis by attenuating nuclear factor-*κ*B (NF-*κ*B) activity [26, 32]. Many studies about natural agents, such as PPAR*γ* agonists, could protect mice from DSS-induced UC through its anti-inflammatory role [33–37]. The activation of the PPAR*γ* response elements (PPRE) in the nuclear is based on forming a heterodimer between PPAR*γ* and retinoid X receptor (RXR). So, agents with synergistic effects on PPAR*γ*/RXR heterodimer may be of better efficacy than pure PPAR*γ* agonists. Interestingly, HKL, apart from its activation of PPAR*γ*, also works as an RXR agonist [38], coincidently meeting this condition.

Besides inflammation, the overwhelming ROS generation and defective antioxidant defense contribute to IBD's progression. Oxidative stress is now regarded as a pathogenic and critical factor in the initiation, progression, and severity of IBD rather than a consequence of chronic inflammation in the intestinal mucosa [39]. NRF2 is a stress-responsive transcription factor associated with cellular homeostasis and has been speculated to have a protective effect on UC by regulating the oxidative stress response and repressing inflammation [40–42]. When the homeostatic balance is interrupted, NRF2 translocates into the nucleus and activates the expression of various genes, one of which is the antioxidant enzyme: HO-1 [43]. Our results showed the activation of NRF2/HO-1 expression in MCEC and the translocation of NRF2 from the cytoplasm to nuclear in RAW264.7 macrophages when treated by HKL, indicating the antioxidant effects of HKL. As previously described, there is a cross-talk between the AMPK and NRF2 axes, and that AMPK works upstream of NRF2 [44–46]. So the activation of AMPK may have both energy homeostatic maintenance and oxidative clearance roles.

AMPK is a crucial cellular energy sensor [47]. AMPK can promote autophagy, which is a process that maintains intestinal barrier function by regulating tight junction proteins [48]. In addition, AMPK has been demonstrated to enhance intestinal barrier function and epithelial differentiation via promoting caudal type homeobox 2 (CDX2) expression [49]. The previous study shows that mitochondrial deacetylase SIRT3 activates AMPK to enhance macroautophagy and chaperon-mediated autophagy to protect hepatocytes against lipotoxicity [50]; since HKL has an apparent activation on SIRT3, the potential mechanism of the interaction between SIRT3 and AMPK in intestinal epithelial barrier enhancement can be further investigated. Apart from its roles in intestinal barrier maintenance, AMPK activators can reduce macrophage inflammation through various signaling pathways, such as AMPK/MAPK [51], AMPK/SIRT1/NF-*κ*B [52], and AMPK/NRF2/HO-1 [44], which indicates the further investigation of HKL as an anti-inflammation agent through AMPK activation. Above all, the upregulated levels of tight junction proteins in the MCEC monolayer when treated by HKL and in the colons of DSS+HKL-treated mice compared to the DSS-induced subjects may be acted through AMPK/NRF2/HO-1 antioxidant pathway and SIRT3/AMPK energy regulation pathway. The cross-talk between these pathways should be further investigated.

Besides new drugs, advanced colon-targeting delivery systems, which exploit multiple characteristics of the colonic environment and exert their actions in parallel to achieve more robust and reliable site-specific drug delivery, can also be utilized to achieve better therapeutic effects [53]. This system can be used to realize the targeted delivery of HKL to the colon focal zone for a better therapeutic action of UC in the future.

## 5. Conclusion

**Figure 9:**
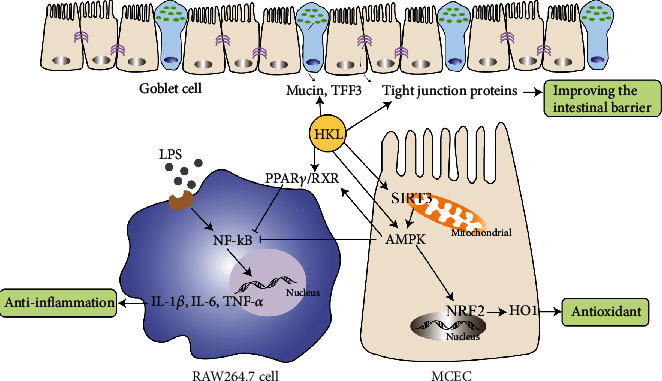
Mechanism of HKL on inhibiting inflammation and improving intestinal barrier in the DSS-induced colitis model.

In conclusion, the present study demonstrated that HKL could alleviate DSS-induced colitis by reducing inflammation, inhibiting oxidative stress, and strengthening intestinal barrier integrity. The underlying signaling pathways likely involve PPAR*γ*/NF-*κ*B, NRF2/HO-1, and SIRT3/AMPK (Figure 9). The noticeable alleviating effects suggest that HKL is a promising treatment strategy for the prevention of UC.

## Figures and Tables

**Table 1:** Scoring system for calculating DAI based on weight loss, stool consistency, and the degree of intestinal bleeding.

Score	Weight loss	Stool consistency	Blood
0	None	Normal	Negative hemoccult
1	1-5%	Soft but still formed	Negative hemoccult
2	6-10%	Soft	Positive hemoccult
3	11-18%	Very soft, wet	Blood traces in stool visible
4	>18%	Watery diarrhea	Gross rectal bleeding

**Table 2:** Scoring system for inflammation-associated histological changes in the colon.

Score	Inflammation	Crypt damage	Ulceration	Edema
0	No infiltrate	None	None	Absent
1	Occasional cells limited to submucosa	Some crypt damage, spaces between crypts	Small, focal ulcers	Present
2	Significant presence of inflammatory cells in submucosa limited to focal areas	Larger spaces between crypts, loss of goblet cells, some shortening of crypts	Frequent small ulcers	
3	Infiltrate is present in both submucosa and lamina propria, limited to focal areas	Large areas without crypts, surrounded by normal crypts	Large areas lacking surface epithelium	
4	A large amount of infiltrate in the submucosa, lamina propria, and surrounding blood vessels, covering large areas of mucosa	No crypts		
5	Transmural inflammation (mucosa to muscularis)			

**Table 3:** Primers used for qRT-PCR.

Gene	Primer sequence	
IL-1*β*	F: 5′-GTTCCCATTAGACAACTGCACTACAG-3′	R: 5′-GTCGTTGCTTGGTTCTCCTTGTA-3′
IL-6	F: 5′-CCAGAAACCGCTATGAAGTTCC-3′	R: 5′-GTTGGGAGTGGTATCCTCTGTGA-3′
TNF-*α*	F: 5′-CCCCAAAGGGATGAGAAGTTC-3′	R: 5′-CCTCCACTTGGTGGTTTGCT-3′
ZO-1	F: 5′-GACCTTGATTTGCATGACGA-3′	R: 5′-AGGACCGTGTAATGGCAGAC-3′
Occludin	F: 5′-ACACTTGCTTGGGACAGAGG-3′	R: 5′-AAGGAAGCGATGAAGCAGAA-3′
Claudin-1	F: 5′-AGGTCTGGCGACATTAGTGG-3′	R: 5′-CGTGGTGTTGGGTAAGAGGT-3′
*β*-Actin	F: 5′-GTCAGGTCATCACTATCGGCAAT-3′	R: 5′-AGAGGTCTTTACGGATGTCAACGT-3′
iNOS	F: 5′-GAACTGTAGCACAGCACAGGAAAT-3′	R: 5′-CGTACCGGATGAGCTGTGAAT-3′
COX2	F: 5′-CAGTTTATGTTGTCTGTCCAGAGTTTC-3′	R: 5′-CCAGCACTTCACCCATCAGTT-3′

## Data Availability

The data used to support the finding of this study are included within the article, and all data are available.

## Abbreviations

UC: Ulcerative colitis
HKL: Honokiol
DSS: Dextran sulfate sodium
LPS: Lipopolysaccharide
MCEC: Epithelial cells
DAI: Disease activity index
IL-1*β*: Interleukin 1*β*
IL-6: Interleukin 6
TNF-*α*: Tumor necrosis factor *α*
iNOS: Inducible nitric oxide synthase
COX2: Cyclooxygenase-2
PPAR*γ*: Peroxisome proliferator-activated receptor *γ*
NF-*κ*B: Nuclear factor-*κ*B
SIRT3: Sirtuin3
AMPK: Adenosine 5′-monophosphate-activated protein kinase
NRF2: Nuclear factor erythroid 2-related factor 2
HO1: Heme oxygenase 1
SPF: Specified pathogen free
HE: Hematoxylin and eosin
DAB: Diaminobenzidine
DMEM: Dulbecco's Modified Eagle Medium
FBS: Fetal bovine serum
CCK-8: Cell Counting Kit-8
TEER: Transepithelial electric resistance
DAPI: 4′,6-Diamidino-2-phenylindole
SDS: Sodium dodecyl sulfate
PVDF: Polyvinylidene difluoride
TBST: Tris-buffered saline Tween
qRT-PCR: Real-time quantitative polymerase chain reaction
TFF3: Trefoil factor-3
PPRE: PPAR*γ* response elements
RXR: Retinoid X receptor
CDX2: Caudal type homeobox 2.
